# Oxaliplatin-induced peripheral neuropathy risk factors and management in Tunisian population

**DOI:** 10.11604/pamj.2020.35.83.18357

**Published:** 2020-03-19

**Authors:** Aref Zribi, Sonia Ben Nasr, Syrine Hamdi, Jihen Ayari, Sana Fendri, Mehdi Balti, Abderrazek Haddaoui

**Affiliations:** 1 Faculté de Médecine de Tunis, Department of Medical Oncology, The Military Hospital of Tunis, Universiy of Tunis El Manar, Montfleury, Tunis, Tunisia

**Keywords:** Chemotherapy, toxicity, oxaliplatin, neuropathy

## Abstract

The most important limits of oxaliplatin treatment is its peripheral neurotoxicity. The aim of our study was to evaluate the oxaliplatin-induced peripheral neuropathy, its impact on treatment and its management. One hundred chemo-naive patients treated with oxaliplatin-based regimen in the medical oncology department of the military hospital of Tunis between 2012 and 2017 were recruited retrospectively. Evaluation of neuropathy was done according to the National Cancer Institute Common Terminology Criteria for Adverse Events (NCI-CTCAE V4). Fifty-six patients were aged more than 60 years. The sex-ratio was 1.56. Twenty-seven patients were overweight, 17 were obese and 56 had a BMI inferior to 25 kg/m^2^. Two patients were consuming alcohol. Twenty-three patients had diabetes. Sixty-four patients developed chronic peripheral neuropathy because of oxaliplatin (grade 1-2 in 58 cases and grade 3 in 6 cases). Sex, BMI, diabetes and alcohol consumption were not associated with the development of peripheral neuropathy. No association was found between grades of neuropathy and sex, alcohol consumption and diabetes. The median cumulative dose of oxaliplatin that induced neuropathy was 432.4 mg/m^2^. The most prescribed treatment was gabapentin (81%) and carbamazepine (16.8%). The treatment was not sufficient to stop neuropathy in 82.6% of cases. Dose reduction was done in 64.2% of cases, treatment delay in 10.7% of cases and treatment interruption in 10.7% of cases. We didn't find any association between known risk factors and peripheral neuropathy. The cumulative dose is interesting to define or to predict the timing of neurotoxicity.

## Introduction

Oxaliplatin is a third generation platinum-based agent largely used in oncology. It's effective in the treatment of localized and metastatic gastrointestinal cancers and has improved survival with a 5 year disease free survival of 78% [1-4]. One of the most important limits of oxaliplatin treatment is its peripheral neurotoxicity. Acute and chronic neuropathy differ in their timing, duration and symptomatology. Acute oxaliplatin neurotoxicity is induced by cold and is characterized by distal sensory symptoms such as paresthesia and dysesthesia occurring in days following oxaliplatin infusion [5]. It occurs rapidly in nearly all patients treated and is typically transient [6,7]. The chronic form occurs because of the repetition of chemotherapy (CT) cycles. Neuropathy is cumulative dose-dependent and can persist for months leading to quality of life deterioration [8-10]. Currently, there is no effective strategy for preventing oxaliplatin-induced peripheral neuropathy (OIPN) and pharmacologic management is limited [11]. The aim of our study was to evaluate the OIPN, its impact on treatment and to discuss its management.

## Methods

**Study population:** one hundred and six chemo-naive patients treated in the medical oncology department of the military hospital of Tunis between 2012 and 2017 were recruited retrospectively. We excluded four patients because of allergy to oxaliplatin and two others because of incomplete data. Patients received an oxaliplatin-based regimen: FOLFOX (5-fluorouracil, leucovorin, irinotecan and oxaliplatin), FOLFIRINOX (5-fluorouracil, leucovorin, irinotecan and oxaliplatin), GEMOX (gemcitabine and oxaliplatin) or XELOX (capecitabine and oxaliplatin). Evaluation of neuropathy was done according to the National Cancer Institute Common Terminology Criteria for Adverse Events: NCI-CTCAE V4.

**Data collection:** we collected parameters that might be associated with neuropathy according to literature: diabetes, alcohol consumption, body mass index (BMI) and neuropathy treatment.

**Statistical analysis:** statistical analysis was performed with SPSS software version 20. The comparison between the different variables was made using the khi-2 test for qualitative variables and the T-student test or Anova for quantitative variables. A p-value equal or less than 0.05 was considered statistically significant.

## Results

Fifty six patients (56%) were aged more than 60 years. Sex-ratio was 1.56. Twenty seven patients (27%) were overweight (BMI between 25 and 30 kg/m^2^), 17 (17%) were obese (BMI superior to 30 kg/m^2^) and 56 (56%) had a BMI inferior to 25 kg/m^2^. Two patients (2%) were consuming alcohol. Twenty three patients (23%) had diabetes (Table 1). Sixty four patients (64%) developed chronic peripheral neuropathy because of oxaliplatin. Thirty five patients (35%) developed grade 1 toxicity, 23 (23%) developed grade 2 toxicity and 6 (6%) had a grade 3 neuropathy. Sex (38% in male and 26% in female, p=0.656) and body mass index (p=0.082) were not associated with the development of peripheral neuropathy. Diabetes (18% in diabetic and 26% in non-diabetic patients, p=0.970) and alcohol consumption (62% if alcohol consumption and 26% if not, p=0.535) were not associated with neuropathy. No correlation was found between grades of neuropathy and sex, alcohol consumption and diabetes (Table 2). The median cumulative dose of oxaliplatin that induced neuropathy was 432.4 mg/m^2^. The most prescribed treatment was gabapentin (81.13%), followed by oxcarbazepine (16.8%). In 82.69% of cases, the medication was not sufficient to stop neuropathy which contributed to dose reduction in 64.2% of cases, treatment delay in 10.7% of cases and treatment interruption in 10.7% of cases.

**Table 1:** Patients characteristics

Variables	Interval	Number
Age	>60 years	56
	≤60 years	44
Sex	Male	61
	Female	39
BMI (kg/m²)	<25	56
	25-30	27
	>30	17
Alcohol consumption	Yes	2
	No	98
Diabetes	Yes	23
	No	77

This table reported the patients characteristics

**Table 2:** Factors associated with neuropathy

Variable		Neuropathy		p-value
		Yes (%)	No (%)	
Sex	H	38	23	0.656
	F	26	13	
Alcohol Consumption	Yes	2	0	0.535
	No	62	36	
Diabetes	Yes	18	10	0.970
	No	46	26	
BMI (kg/m²)		27.47±4.91	23.81±3.73	0.082
Age (years)		58.59±10.89	61.86±9.746	0.062

This table reported the risk factors associated with neuropathy

## Discussion

In our population, 64% of patients developed chronic peripheral neuropathy. Berg C *et al.* reported a rate of 71% of neuropathy [12]. According to our study, neither age, sex, alcohol consumption nor BMI were associated to neuropathy. We didn't find any association between known risk factors and OIPN probably because the study was retrospective with a limited number of patients. A study done by Shahriari A *et al.* [13] on patients with colorectal cancer in Iran reported no association between age, sex, alcohol consumption and incidence of neuropathy. Patients with neuropathy had a higher BMI (p=0.003). Incidence of neuropathy was higher if alcohol consumption was ≥5 glasses in a single occasion for men and ≥4 glasses in a single occasion for women, usually within 2 h (p=0.003) [7] and grade 2/3 neurotoxicity occurred more often if there was high alcohol intake [14]. Diabetes wasn't associated with neuropathy in our study. Uwah AN *et al.* [15] evaluated the relationship between preexisting diabetes and OIPN and found a mean cumulative dose at onset of oxaliplatin regimen of 554 mg/m^2^ for all patients with neuropathy, 388 mg/m^2^ for patients with diabetes and 610 mg/m^2^ for patients without diabetes. Although patients with diabetes developed OIPN at a lower cumulative dose of oxaliplatin, diabetes did not appear to affect the severity of OIPN. Other troubles such as hypomagnesaemia and anemia must be considered as they seem to be associated with peripheral neuropathy. These biological disorders are easily measurable before the initiation of treatment and could predict toxicity.

There is an association between the incidence of neuropathy and hypomagnesaemia and anemia [13]. The cumulative dose is interesting to know to prevent the risk of treatment discontinuation. The chronic form of OIPN is cumulative dose-dependent characterized by distal sensory symptoms causing functional impairment in approximately 15% of cases receiving a 780-850 mg/m^2^ cumulative dose of oxaliplatin [8-9]. Berg C *et al.* retained a median warning threshold of 600 mg/m^2^. This defined threshold will alert the doctor of the risk of continuing treatment [12]. According to our study, the average cumulative dose that induced neuropathy (all grades included) was 432.4 mg/m^2^. The average cumulative dose found in the Tunisian population is inferior to that of other studies done in other populations. Oxaliplatin-induced peripheral neuropathy (OIPN) may vary in frequency and severity among different cancer patients despite equal treatment schedules. A genetic susceptibility for more severe OIPN should be discussed in more researches to investigate the molecular mechanisms of oxaliplatin neurotoxicity. The chronic form of OIPN worsens quality of life and hampers the long-term administration of oxaliplatin, leading to dose reduction, treatment delay or discontinuation of treatment. Neuropathy could be reduced by the use of medication, but in our study only 17% of patients responded to treatment. Argyriou *et al.* conducted a randomized, open-label, controlled trial to assess the efficacy of oxcarbazepine for OIPN treatment.

Thirty-two patients with colon cancer received 12 courses of the FOLFOX-4 regimen and were randomly assigned to receive oxcarbazepine (600mg BID) or CT without oxcarbazepine. The incidence of OIPN was lower with oxcarbazepine (31.2% vs 75%) [16]. Magnowska M *et al.* evaluated the response to gabapentin in a group of patients who developed neuropathy. There was an improvement with gabapentin in symptoms (p<0.027), pain (p<0.027) and neurologic deficit (p<0.019) [17]. Yang *et al.* showed evidence of duloxetine (a balanced selective serotonin (5-HT)) and norepinephrine reuptake inhibitor (SNRI) efficacy in chronic OIPN treatment in patients with colorectal cancer after oxaliplatin-containing CT. Duloxetine effective dose was 60 mg/day [18]. Calcium and magnesium infusion before CT initiation is a routine preventive action. However, its effectiveness remains controversial [19]. A randomized phase III study on 353 patients treated with Folfox showed that there is no statistically significant difference in the incidence of neuropathy between magnesium and calcium infusion and placebo [19], while a retrospective study on 102 patients under FOLFOX regimen, reported that magnesium and calcium infusion decreased the incidence of chronic neuropathy [20]. Balaysac *et al.* demonstrated the preventive effect of a polyamine reduced diet on both acute and chronic OIPN [21]. According to the latest American Society of Clinical Oncology (ASCO) recommendations, infusion of magnesium and calcium solution to prevent neuropathy it is no longer recommended. Use of duloxetine is recommended because of its proven efficacy in many researches [22].

## Conclusion

In our study, we didn't find any association between known risk factors and OIPN. The cumulative dose is interesting to define or to predict the timing of neurotoxicity. We may then consider protocol continuation, doses re-estimation or therapeutic alternative, benefit/risk ratio evaluation. Few ways to prevent and limit neurotoxicity are yet available.

### What is known about this topic

- OIPN can limit treatment with oxaliplatin;
- The chronic form of OIPN is cumulative dose-dependent, cumulative dose of oxaliplatin reported in the literature was between 600 and 850mg/m^2^;
- There are no agents recommended for the prevention of OIPN.

### What this study adds

- Our Tunisian population was characterized by a cumulative dose of oxaliplatin inducing OIPN below the dose described in the literature;
- Tunisian patients are more vulnerable to oxaliplatin than other patients probably for genetic reasons;
- OIPN worsens quality of life and disrupts the course of treatment with oxaliplatin.

## Competing interests

The authors declare no competing interests.
